# Case Formulation in Young People with Post-Traumatic Stress Disorder and First-Episode Psychosis

**DOI:** 10.3390/jcm5110106

**Published:** 2016-11-23

**Authors:** Emma Halpin, Vanessa Kugathasan, Carol Hulbert, Mario Alvarez-Jimenez, Sarah Bendall

**Affiliations:** 1 Headspace Youth Early Psychosis Program, Alfred Health, Prahan, Victoria 3181, Australia; e.halpin@alfred.org.au; 2 Psychological Sciences, The University of Melbourne, Melbourne 3052, Australia; v.kugathasan@student.unimelb.edu.au (V.K.); cah@unimelb.edu.au (C.H.); 3 Orygen, The National Centre of Excellence in Youth Mental Health, and the Centre for Youth Mental Health, University of Melbourne, Parkville 3052, Australia; Mario.Alvarez@orygen.org.au

**Keywords:** case formulation, first-episode psychosis, post-traumatic stress disorder, cognitive behavioural therapy

## Abstract

Background: Evidence based treatment interventions for young people with first-episode psychosis (FEP) and trauma histories is lacking. Although case formulation (CF) has been widely regarded in cognitive behavioural therapy manuals as beneficial, there is limited empirical research examining how clients and therapists experience the process. Aim: This study aimed to explore young people’s reactions to CF in treatment for PTSD (post-traumatic stress disorder) and FEP. Method: Semi-structured interviews were conducted with three participants (aged 19–20) with FEP and PTSD and their therapists, after they had completed a trauma-focused treatment program with a CF component. Transcripts were analysed using an interpretative phenomenological approach and themes were derived. Results: Two themes related to participants’ experiences were identified from the analysis: (1) Developing Insight; (2) A challenging experience; and two themes from the therapists: (1) Doing the case formulation; (2) Value of case formulation. Participants and therapists reported benefits in making connections between current symptoms and past trauma. Participants viewed the process as challenging. Conclusion: Results suggest a potential discrepancy between the experience of the case formulation process for clients and therapists.

## 1. Introduction

The relationship between trauma and psychosis is now well established [1,2,3]. Childhood trauma has been suggested as an aetiological factor in the development of psychosis [4,5], and people with psychosis are more likely to be exposed to trauma throughout their lives [6] and report traumatic symptomatology as a consequence of their illness [7]. The impact of trauma on psychotic symptomatology and overall functioning is widely considered to be detrimental, with individuals showing higher levels of depression and anxiety [8,9,10], positive symptoms and cognitive symptoms [9,10] and reduced participation in vocational rehabilitation [11].

Despite the recognised need for specialised therapeutic programs [12], there is limited empirical data on the treatment of trauma and psychosis. Several trials have aimed to treat PTSD (post-traumatic stress disorder) symptoms in individuals with psychosis [13,14,15,16], but have not specifically aimed to address both the effects of PTSD and comorbid psychosis. For example, van den Berg and colleagues examined the impact of eye movement desensitization and reprocessing (EMDR) and prolonged exposure to reduce PTSD symptoms in 108 participants with psychosis and PTSD [16]. Participants were more likely to achieve loss of PTSD diagnosis during both treatments than those in the waitlist control. Furthermore, there have been few studies examining treatment for individuals with their first episode of psychosis (FEP). 

Alongside specific interventions directly targeting PTSD in FEP, there is a widely identified need for a broad, service-wide understanding of, and intervention for, trauma-exposed mental health consumers that goes beyond the treatment of PTSD [17,18,19,20]. One name coined for this is “trauma-informed care”, a care model that is gaining traction across the world [18,19]. A major barrier to the implementation of trauma-informed care is a lack of evidence to support the model [20], which may stem from a lack of clear operationalisation [17]. There is a need in psychosis treatment services for both trauma-specific and trauma-informed approaches [21]. Further, FEP clinicians have concerns that assessment of and interventions for trauma may trigger traumatic memories or psychotic symptoms, and this deters them from assessment and treatment of the effect of trauma [22].

A comprehensive case formulation (CF) is considered to be a cornerstone of cognitive behavioural therapy (CBT) [23,24]. CF is identified in CBT manuals as having particular utility in situations where empirically supported treatment protocols are insufficient [25]. Undertaking a CF involves forming a hypothesis about the psychological underpinnings of a patient’s difficulties, highlighting factors that may have caused, precipitated and maintained these difficulties [20]. It is generally conceptualised as a collaborative endeavour between therapist and patient [23,24], and often forms the basis for the development of an individualised treatment plan [26]. There is high utility in using CF in both a trauma-informed and a trauma-specific intervention in FEP, as both models stress the importance of an understanding of the role of the trauma and peri-traumatic effects on presenting symptoms [12,17].

Despite theoretical acceptance of the importance of undertaking a CF, there has been little empirical examination of its unique contribution [27,28]. Inter-rater reliability between therapists of formulations is fairly low, suggesting differences between therapists in beliefs regarding what is essential to CF [29,30]. Client reactions to the process are mixed, with studies reporting increased insight into their difficulties, a stronger therapeutic relationship and less self-blame, coupled with pessimism, emotional pain, or feeling that aspects of the process were unhelpful, confusing or limiting [19,31,32,33]. 

The present study sought to explore the experience of CF by examining the client and therapist experiences of formulation in a CBT-based case-management intervention for symptoms of PTSD and psychosis in those with a FEP. The intervention was designed in collaboration between researchers and clinicians in an Australia FEP service in order to meet the pragmatic needs of ‘real-world’ clinical practice. It was specifically tailored to be able to be implemented by clinicians with a wide variety of skills and experiences rather than cognitive-behavioural specialists, as this is the workforce that typically treats young people with FEP. It was also designed to be highly flexible and to address concerns regarding symptom exacerbation in order to facilitate its use in clinical practice. This intervention was developed and piloted rather than using an existing CBT treatment for either psychosis or PTSD, as those treatments would be difficult to implement within the Australia service delivery model. However, the intervention does utilise elements common in evidence-based PTSD therapies [16,34,35,36]. The study took an exploratory approach, using Interpretative Phenomenological Analysis (IPA) [34]. IPA methodology was selected to examine how participants make sense of and understand a particular experience that has strong personal significance [37]. The aim of the study was to inform the development of effective treatments for those with trauma histories and FEP.

## 2. Experimental Section

### 2.1. Participants and Context

Three participants were recruited from a pilot trial of a newly-developed intervention to treat the symptoms of trauma for young people with FEP [38], within the Early Psychosis Prevention and Intervention Centre (EPPIC) at Orygen Youth Health, a publically funded mental health program providing intensive outpatient treatment for young people aged 15–25 with emerging psychotic disorders in Melbourne, Australia. The trauma intervention was integrated into routine case management tasks [39] and involved an assessment and treatment phase. The intervention treatment manual is not yet publicly available and therefore a detailed description of the relevant modules will be described here. The assessment phase included four modules that will be described: 1. Coping, 2. Assessment, 3. Psychoeducation and 4. Formulation. The treatment phase will not be discussed as it does not pertain to the current study. The modules were delivered in a fixed order but the number of, and how sessions were delivered in relation to other case management tasks, was flexible and determined by the needs of the young person. 

The assessment phase began with the (1) Coping module. This consisted of: skills development for noticing and communicating in-the-moment distress levels (using subjective units of distress (SUDS)); learning and practice of distress coping strategies for use in and out of therapy; and assessment and treatment of safety concerns such as suicidality, self-harm and substance abuse. Once distress coping skills were mastered, the (2) Assessment and (3) Psycho-education modules began in parallel. The timing and titration of these modules depended on the client’s level of distress in session and was measured by SUDS ratings. The assessment module involved the development of a written timeline of major life experiences including trauma, and the onset and development of PTSD, depressive, dissociative and psychotic symptoms, suicidality and reduced functioning. While not designed as exposure treatment, it has been suggested that comprehensive assessment of trauma can act as covert exposure in people with PTSD and psychosis [16]. The psycho-education module involved education about the symptomatic effects of trauma including PTSD and dissociative symptoms. The final assessment module, (4) Formulation, lasted between 1 and 2 sessions. The relationship between the trauma experienced and the development of subsequent symptoms was explored, based on the knowledge the young person had gained through psycho-education. This was developed into a formulation letter. Subsequent treatment modules were implemented based on the formulation.

Inclusion criteria for the study were (a) a psychotic disorder or mood disorder with psychotic features, diagnosed using the criteria of the Diagnostic and Statistical Manual of Mental Disorders (4th Edition, DSM-IV); (b) aged between 15 and 25 years; and (c) a current diagnosis of PTSD at (assessed using the Clinician Administered PTSD Scale (CAPS-IV) for both most severe past trauma and acute psychotic episode [40]); Exclusion criteria were: (a) IQ less than 70; and (b) inability to speak English. Participants’ diagnoses and scores on relevant psychometric measures are shown in Table 1. Two therapists, both clinical psychologists, took part in this study. Therapist 1 (undertook CF with participant 1) had one year of therapy experience post study (Doctorate level), therapist 2 (participants 2, 3) had 16 years’ experience (Masters level).

### 2.2. Procedure and Measures

The Structured Clinical Interview for DSM-IV [41] was used to diagnose primary psychotic disorder and PTSD symptoms were measured using the CAPS [40].

The formulations created with each client involved collaboratively compiling a highly individualised explanation for the development of the presenting and past symptoms (including PTSD, hallucinations, delusions and/or dissociation) in relation to the trauma experienced. The “5 P” approach, encompassing the presenting problem, predisposing factors, precipitating factors, perpetuating factors and protective factors, was one of the tools used [22], and the information was based on that gathered in the assessment phase. Particular emphasis was put on assisting the clients to view their symptoms in relation to their life experiences, such that, in some cases, clients were asked to remember and reflect on past avoided experiences. There was no time limit on sessions for the CF, as it was unclear what length of time would be needed to formulate potentially multiple presenting problems in this group. One to two sessions was the guideline. It is a flaw of the research that we do not have the specific number of sessions for each participant.

The goal of the module was to have a written formulation in the form of a letter that was agreed upon. Participants were invited to write their own letter and share it with their therapists if they wished. This was considered an important aid to collaboration, and to allow young people to be aware of what information about trauma was in their clinical file. At the completion of the formulation, participants were asked by their therapist if they would like to participate in a sub-study. Formal written consent was gained from both participant and therapist. Participants 1 and 2 were interviewed approximately six months post CF, participant 3 approximately one month post CF. This time difference was due to the difficulties recruiting participants for the study. Therapists were interviewed in the same week as the participant interview. 

The interview followed a semi-structured interview schedule developed by the research team following guidelines on IPA [34]. The interviews lasted approximately one hour each. To specifically target the impact of the CF, the interviewer used specific language, including “what was it like talking about different times in your life”, “what is your understanding of your problems now”, “what is your understanding of trauma in your life”, “what have you taken from your understanding of your problems”, and asked about the formulation letter with participants. Interviews with therapists used clinical language (e.g., “what was it like completing the formulation”). Interviews were audio-taped and transcribed verbatim. Participants were reimbursed $30.

Ethical approval was gained from the Melbourne Health Mental Health Research and Ethical Committee and the University of Melbourne Ethics Committee. 

### 2.3. Data Analysis

In line with the framework for IPA analysis suggested by Smith et al. (2009) [34], analysis initially involved close reading of each individual transcript to explore the semantic content and language used by each participant. Emergent themes from each transcript were then drawn out based on concrete verbatim data. Connections between emergent themes (within each individual transcript) were made to develop a set of super-ordinate themes. Comparisons were then made across cases to identify shared thematic patterns. Analysis was grounded in concrete, verbatim data extracts from the recorded interviews to illustrate the themes. Reflexivity within the coder was encouraged via discussion with the research team, and the use of a reflexive diary [42]. Guidelines for publication of qualitative studies [43] were followed to ensure research credibility.

## 3. Results

### 3.1. Participant Interviews

As the participants in the study were asked broad questions about their understanding of the relationship between their traumas and the current problems (in order to access their understanding of their formulation), as well as specific questions about the formulation process, they described many aspects of the therapy which were not directly related to formulation but to the larger intervention. These were categorised under the theme headings of: the powerfulness of trauma-related symptoms; long-standing nature of problems; treatability of problems; getting better; belief in self, feeling comfortable with therapist; feeling heard; my opinion is important. These themes are likely to have been a part of the experience of CF. However, it was not possible to isolate them from the other therapy modules. Therefore, only the themes directly relating to the CF process are described here. One of these had subordinate themes (see Table 2).

#### 3.1.1. Developing Insight

All three participants described the way in which CF involved making links between the experience of past traumatic events and the emergence of present symptoms and ways of being.

“I have abandonment issues. So we were kind of going back through my life to see, like, what caused them.”

*—P1*

”I talked about how the psychosis, the voice and the images in my head would be about my past.”

*—P2*

Frequent mention was made of using the timeline to determine when certain symptoms occurred in relation to traumatic events, with reference to how this information was compiled into the formulation. Participants also demonstrated an understanding of what precipitated their problems in the present day and what maintained them.

“I would have to go to the Plaza again and then the thoughts would come… She even wrote on the whiteboard how the vicious cycle happens.”

*—P2*

“Pretty much my depression is triggered by anything relating to friends or family.”

*—P1*

Two participants also described their awareness of the way in which avoidance can perpetuate their difficulties and described using actions such as mindfulness to prevent these problems from escalating.

“I learnt through [therapist name] and the letter that it’s alright to feel scared and stuff but that I have to let it go and just think alright if I don’t do this then I’ll regret it later.”

*—P2*

“I can see that that’s what’s triggered me so I can calm down easier… In the past I wasn’t able to do that and I would just get overwhelmed and stuck.”

*—P3*

Participant 3 described gaining insight into the development of connections but did not feel that making those connections was helpful. She reported that she wanted to spend more time on present symptoms. She expressed that she felt that her therapist was dismissive of her feelings, preferring to move into CF rather than continue discussing her difficulties with rage. This client also expressed annoyance at inaccuracies in the CF letter.

“It did help highlight where it all stemmed from but it doesn’t feel like it’s really helped me much… It didn’t feel relevant to what I’m going through now.”

*—P3*

#### 3.1.2. A Challenging Experience

This theme arose from participants’ reflections on the difficult nature of completing a CF. Participant 1 noted that she was unable to complete her own written CF due to the overwhelming distress when she began speaking about her trauma history. Her therapist also did not complete the letter due to level of displayed distress.

“Because I’m trying to put that in the past with XX beating me and stuff like that and when it [CF] got up to XX I’m just like I can’t.”

*—P1*

She recounted the way in which talking about her past appeared to intensify her auditory hallucinations, *“the voices would grow louder”*, and made reference to herself as “*weak*” as she was unable to complete the process. Participant 2 recounted a confluence of emotions when thinking of her past trauma with this getting progressively more difficult as the therapist moved to more traumatic events. Participant 3, whilst on the one hand describing the experience as “*unemotional*”, also recalled disassociating in session and feeling tiredness post session, suggesting an emotional impact of the experience.

### 3.2. Therapist Interviews

Two superordinate themes emerged from the analysis of therapist interviews. Both had related subordinate themes (see Table 3).

#### 3.2.1. Doing the Case Formulation

Therapists characterised the process as involving making connections between the past and present as a way to understand the participant’s present situation.

“Drawing those events together and perhaps finding meaning in them and realising oh maybe they weren’t so coincidental and what was happening there?”

*—T1*

The metaphor of a puzzle emphasised the way in which CF was a process of creating a whole from disparate seemingly unconnected parts and seeing a meaningful picture emerge.

“It wasn’t until we kind of glued them together that we really looked at the whole kind of narrative and it is then that she looked at the patterns.”

*—T2.1*

The process of gaining insight was described as exploratory, with therapists often not sure of the final outcome or how they might arrive there. Therapists spoke of going on a tangent and being led by the participant. Therapist 2 reflected on the way in which these “*side roads*” were often more fruitful as a means for understanding what was important to the participant.

#### 3.2.2. Value of Case Formulation

Therapists expressed that the CF was powerful and was akin to undertaking an intervention with the participant, particularly when done collaboratively.

“The formulation at times might be the therapy”

*—T2.1*

“I think through the process they are reprocessing a lot of the memories”

*—T2.2*

Therapists described the way in which undertaking the process elicited changes in the participants’ lives and interpersonal patterns. Both therapists reported that they had gained a better understanding of their participants through undertaking the CF, with therapist 1 reporting that she was able to know the participant “*beyond just her diagnosis*”. This was likened to peeling away layers of an onion, suggesting that the CF allowed the therapist to see the participant on a deeper level beyond the surface issues. In the use of this metaphor, the therapist also highlighted the messiness of this process of peeling away to get to that stage. 

Therapist 1 commented on the difficulties her client (P1) experienced undertaking the CF, questioning whether her client felt able to say she did not want to continue with the process.

“She was really avoidant about those life experiences because they were still traumatic obviously… So I think for me I could yeah, sense that avoidance [of the CF] really quite early on.”

*—T1*

“I do really believe that if we’re going to do something it’s got to be meaningful for the person in the room… So that’s what I was concerned about. That she wasn’t able to find that voice to be able to say actually that isn’t meaningful for me or I’m not really sure.”

*—T1*

## 4. Discussion

### 4.1. Summary of Findings

Participants and their therapists made reference to the value of making connections between participants’ current symptoms and their past trauma history, with a particular focus on maintaining factors for their distress. Both described the process as challenging, and therapists focused on the need to be guided by clients’ needs and the importance of going slowly. 

### 4.2. Developing Insight

Therapists reported that undertaking the CF afforded them insight into the predisposing, precipitating and perpetuating factors of their clients’ symptoms, in line with manuals advocating the benefits of undertaking a CF [44,45,46]. Participants noted the connections drawn between their past trauma and current symptoms. For two of the participants, this insight allowed them to make relevant changes to ameliorate the perpetuation of their symptoms, highlighting the importance of illustrating maintaining factors in a formulation [32,33]. Therapists reported a greater knowledge of their clients’ difficulties and described seeing clients beyond their presenting symptoms, allowing them to be more sensitive to factors that may affect the therapeutic process. Indeed, Horowitz and colleagues (1989) proposed that a CF helps the therapist to understand the nature of the therapeutic relationship and thus elicit greater empathy for the client beyond the presenting problems [47] (see also [31,48]).

Although therapists found the process of making connections between the past and present as helpful, participants had more mixed reactions. One participant described feeling dismissed by her therapist, wanting to focus more on current symptoms. This may highlight a difference between client and therapist in their perceptions of the utility of a formulation—while, for clients, the development of insight may be somewhat helpful but not contribute directly to symptom alleviation, for therapists it may be essential for treatment planning, especially in areas with no available evidence-based treatments. It may be helpful to more explicitly discuss this aspect of CF with clients so that they have a clear rationale for undertaking a CF. As noted by Therapist 1, there needs to be a focus on what is “meaningful” for the client.

It was evident that in both the therapists’ and participants’ descriptions of the process, they were reflecting on both the 1–2 sessions of formal CF and the ongoing assessment of events and symptoms within the larger assessment phase. This is understandable as psychoeducation was also delivered during this assessment phase, which involved education about the symptomatic effects of trauma (e.g., reappraisal of past symptoms).

### 4.3. Participant Distress

Participants spoke of the challenges of the process of formulating. One participant found the process of revisiting memories as part of CF so difficult that she was unable to complete aspects of it. She also reported distress completing the timeline component of the assessment module. Another described it as difficult, while the third reported feeling overwhelmed, experiencing disassociation in-session and exhaustion after CF sessions. Both therapists recognised the difficulties experienced by their clients. This is despite the CF being delivered post, and in conjunction with, a coping module intended to build the participants’ emotion regulation skills. The results may thus suggest that this coping module did not fulfill its role for some participants. The therapy may require a more comprehensive safety module with specific attention on facilitating the ability of the client to communicate distress to the therapist.

While not designed as exposure treatment, it has been suggested that comprehensive assessment of trauma can act as covert exposure in people with PTSD and psychosis [16]. Therapist 2 reported that her client ‘reprocessed’ the memories while undertaking the CF. Studies investigating the impact of revisiting trauma in primary PTSD treatment have also found distress, worsening of PTSD symptoms, and emotional exhaustion during and immediately after trauma therapy sessions [49,50,51,52]. These studies asked participants to reflect on the whole of their trauma therapy, rather than the formulation process. As such, it is not clear when in the therapy process participants experienced these reactions. However, the cognitive model of PTSD [53] predicts distress during the memory-processing phase of therapy. In the current study, distress is clearly present earlier in the therapeutic process. This was likely due to the requirement of the idiosyncratic formulations—more information about the trauma and its effects needed to be gathered than in traditional trauma-focused CBT. There are currently no well-defined and evidence-based models with which to scaffold a more brief and less emotionally intense assessment and CF. More research is required to understand the costs and benefits of distress during the therapy process.

It is important to note that on the few occasions that clients with psychosis have been asked about their experience of CF, they describe both positive and negative experiences. Chadwick et al. (2003) [23] found that their participants reported an increased understanding of their presenting issues following formulation, but many also described negative aspects, including that it has been ‘saddening, upsetting and worrying’ (p. 674). 

### 4.4. Limitations

Our study holds a number of limitations. Our sample size was very small, thus limiting the generalisability of the findings. The fact that the CF was not heavily structured and did not occur within a defined number of sessions (compared to past examinations of the CF process such as that by [27]), are both further limitations. As the interviews occurred, in two cases, months post formulation, it was difficult for the interviewer to disentangle the comments relating to the assessment module of the larger intervention from those relating specifically to the CF. All efforts were made to do so. Interviews immediately after the formulation module would have combated this. It is also is notable that the sample comprised only female clients and therapists, and one therapist was interviewed twice. The difference in the level of experience between the two therapists is a further limitation.

## 5. Conclusions

This small qualitative study was designed to explore the subjective experiences of a case formulation in a small group of young adults with post-traumatic stress disorder and a first-episode of psychosis, and their therapists. Results suggest that while therapists found the process essential to their understanding of the client, clients experienced case formulation as useful to identify connections between their past and current symptoms, but also as distressing and challenging. These findings need careful consideration. In clinical practice, it is widely accepted among cognitive behavioural therapists that a comprehensive and valid case formulation is needed to successfully treat a person in distress, even more so when the presenting problems are complex [25,54] and when there are no evidence-based treatments available [55]. Our results support other studies, which show that clients can experience this process as difficult in a number of ways [27,31,32,33]. Our results highlight the need for further research into therapeutic models of trauma and psychosis that will enable us to assess and formulate in a more precise way. They also emphasise the need for research into the experience of therapy, especially in complex and high-risk groups, as clients may have distressing experiences of therapy that they do not share with their therapists.

## Figures and Tables

**Table 1:** Participant characteristics.

Clincial Information	Participant 1	Participant 2	Participant 3
Age	19	19	21
Psychosis diagnosis	Schizophrenia	Schizophrenia	Schizoaffective disorder
Clinician administered PTSD scale event scores	Sexual abuse (58) Psychosis (26)	Bullying (58) Psychosis (50)	Sexual abuse and bullying (101) Psychosis (56)

**Table 2:** Super- and sub-ordinate themes arising from analysis of participants’ transcripts of their experiences of case formulation (CF).

Superordinate Themes	Subordinate Themes
Developing Insight	Making links between past and present
A challenging experience	Awareness of the triggers and maintaining factors of problems

**Table 3:** Super- and sub-ordinate themes arising from analysis of therapists’ transcripts of their experiences of CF.

Superordinate Themes	Subordinate Themes
Doing the case formulation	Making connections Exploring
Value of case formulation	Case formulation as an intervention Getting to know the client better
